# Copy-Number Alterations and Copy-Neutral Loss of Heterozygosity in Adult T-ALL: A Descriptive Study in Relation to Day +70 MRD

**DOI:** 10.3390/ijms27209348

**Published:** 2026-10-21

**Authors:** Natalya Risinskaya, Sofia Starchenko, Abdulpatakh Abdulpatakhov, Yulia Chabaeva, Olga Aleshina, Ekaterina Kotova, Tatiana Obukhova, Anna Yushkova, Olga Maiorova, Nikolay Kapranov, Irina Galtseva, Valeria Surimova, Sergey Kulikov, Andrey Sudarikov, Elena Parovichnikova

**Affiliations:** National Medical Research Center for Hematology, 125167 Moscow, Russia; abdulpatakhov.a@blood.ru (A.A.); chabaeva.y@blood.ru (Y.C.); gavrilina.o@blood.ru (O.A.); kotova.e@blood.ru (E.K.); obukhova.t@blood.ru (T.O.); yushkova.aa@blood.ru (A.Y.); maiorova.o@blood.ru (O.M.); kapranov.n@blood.ru (N.K.); galtseva.i@blood.ru (I.G.); surimova.v@blood.ru (V.S.); kulikov.s@blood.ru (S.K.); sudarikov.a@blood.ru (A.S.); parovichnikova.e@blood.ru (E.P.)

**Keywords:** T-cell acute lymphoblastic leukemia, chromosomal microarray analysis, copy-number alterations, copy-neutral loss of heterozygosity, measurable residual disease, early T-cell precursor ALL, CDKN2A, 9p21.3

## Abstract

Chromosomal microarray analysis (CMA) detects copy-number alterations and copy-neutral loss of heterozygosity (cnLOH), but the CMA landscape of adult T-ALL has been described in few cohorts. Day +70 measurable residual disease (MRD) is a surrogate marker of early treatment response, available only after induction. Thirty-nine adults with Ph-negative T-ALL on a single protocol underwent diagnostic CMA. Mosaic events were catalogued per chromosome and against a 57-gene panel and described in relation to day +70 MRD status (13 MRD-positive, 25 MRD-negative). The analysis is presented as descriptive without formal inference. Mosaic alterations were present in almost all patients, so groups differed in the amount rather than the presence of genomic change. Complex profiles (≥3 affected chromosomes) were seen in 46.2% of MRD-positive and 20.0% of MRD-negative patients. Biallelic 9p21.3 deletion occurred in both groups, whereas 9p cnLOH without deletion occurred predominantly in MRD-negative patients. Total cnLOH burden did not track with copy-number burden. Copy-number and copy-neutral events are mechanistically distinct and were not interchangeable here: 9p deletion and 9p cnLOH are separable by SNP array and should be reported separately rather than pooled. No association reached significance, although the cohort was too small to detect all but very large effects.

## 1. Introduction

T-cell acute lymphoblastic leukemia (T-ALL) is an aggressive leukemia that arises from immature T-cells and accounts for about 20–25% of adult ALL cases [1,2,3,4]. Cytogenetics at diagnosis is traditionally used for risk stratification, but conventional karyotyping has limited prognostic value in T-ALL: in our own cohort, karyotype abnormalities at diagnosis were not associated with 5-year survival [5].

Microarray and sequencing studies have since described many recurrent genetic changes in T-ALL, including alterations of transcription factors and of the *CDKN2A/CDKN2B* cell-cycle genes [2,4]. Several of these changes have been linked to prognosis: for instance, poor outcomes with 17p deletion or *IDH1/IDH2* and *DNMT3A* mutations, and better outcomes with *NOTCH1/FBXW7* mutations or homozygous *CDKN2A/CDKN2B* deletion in some subtypes [4]. The associations are, however, often subtype-specific and rarely strong enough on their own to guide treatment intensity.

What has not been well studied is whether these genomic changes predict how well a patient responds to initial therapy. Measurable residual disease (MRD) after induction is one of the strongest outcome predictors available: in our cohort, MRD positivity on day +70 identifies patients with markedly lower 3-year relapse-free survival (35% vs. 79%, *p* < 0.001) and is used to decide who should proceed to allogeneic transplantation [5].

Day +70 MRD status is itself a surrogate marker of early treatment response, but it becomes available only after induction has been completed. This raised a practical question: Does chromosomal microarray analysis (CMA) at diagnosis reveal copy-number patterns that relate to subsequent MRD status. In other words, could a baseline genomic feature anticipate the surrogate endpoint before it is measurable?

This cohort of 38 evaluable patients is too small to answer that question with statistical rigor. We applied conventional group comparisons and exploratory modeling, but with 13 MRD-positive events, these analyses could not yield reliable estimates, and we therefore present the study as descriptive: we characterize the spectrum of copy-number alterations and cnLOH and describe it in relation to MRD status, without formal inference and without correction for multiple testing.

We examined two types of change detectable by CMA—copy-number alterations (CNAs) and copy-neutral loss of heterozygosity (cnLOH)—first across the genome at a chromosome level and then restricted to loci containing cancer-related genes. For the gene-level analysis, we used a pre-defined list of 57 cancer genes taken from the commercial xGen 57G Pan-Cancer Amplicon Panel (Integrated DNA Technologies, Coralville, IA, USA) and described these gene-level events by MRD status.

## 2. Results

Table 1 summarizes the main clinical characteristics of all 39 patients, split by MRD status at day +70. One patient died before day +70 and is shown in a separate column and excluded from all comparisons between MRD groups.

MRD-negative patients had higher white-cell counts, LDH, and blast percentages in blood and marrow than MRD-positive patients (Table 1). The T-III (cortical) immunophenotype was more common among MRD-negative patients (48.0% vs. 15.4%), while an immature ETP/near-ETP phenotype was more common among MRD-positive patients (46.2% vs. 28.0%). Detailed cytogenetic, FISH, and CMA data for each patient are given in Supplementary Table S1.

An abnormal karyotype was more frequent in MRD-positive patients (8/13, 61.5%) than in MRD-negative patients (11/22, 50.0%), but the difference did not reach significance (*p* = 0.73, Fisher’s exact test).

Bone marrow samples always contain some non-leukemic cells, so we restricted the analysis to mosaic (subclonal) events detected by CMA. All non-mosaic aberrations in our dataset were also captured as mosaic calls at the same loci while the reverse was not true, confirming that mosaic calls give a more complete picture of the leukemic genome. Only mosaic events were therefore carried forward.

CMA detected copy-number alterations or cnLOH in all 13 MRD-positive and 24 of 25 MRD-negative patients (Figure 1 and Figure 2; Supplementary Table S1). The two groups differed mainly in how much genomic change was present, not in whether it was present at all. Complex profiles (≥3 affected chromosomes) were more common in MRD-positive patients (46.2% vs. 20.0%, *p* = 0.13, Fisher’s exact test).

We assessed copy-neutral loss of heterozygosity (cnLOH) in both groups of patients (Figure 2). While 9p deletions occurred in both groups, cnLOH at 9p was seen mainly in MRD-negative patients (6/25, 24.0%) versus MRD-positive patients (1/13, 7.7%; *p* = 0.38).

Among the seven patients with 9p cnLOH, three (2 MRD-negative patients, 1 MRD-positive patients) also had a focal biallelic deletion of 9p21.3 nested inside the cnLOH region (Supplementary Figure S1); in the other four (all MRD-negative patients), the region retained two intact copies. A further six patients had biallelic 9p21.3 deletion without any cnLOH (one MRD-positive patient, five MRD-negative patients; Supplementary Figure S2). In total, nine patients had biallelic 9p21.3 loss (two MRD-positive patients, seven MRD-negative patients), arising either inside a cnLOH segment (three cases) or without cnLOH (six cases). Eight of these nine patients had a non-ETP immunophenotype.

Total cnLOH burden (number of affected chromosomes) did not differ between groups (6.16 ± 4.14 in MRD-negative vs. 5.62 ± 4.05 in MRD-positive patients; *p* = 0.69). Unlike copy-number burden, overall cnLOH burden was similar between groups, while cnLOH at 9p specifically was more frequent in MRD-negative patients, without reaching statistical significance (*p* = 0.38, as above).

The distribution of alterations across the genome is shown in Figure 3. Copy-number changes were spread across most chromosomes in both groups, without a dominant recurrent locus.

Exploratory chromosome-level feature-selection analyses (penalized regression and random forest) were applied to a matrix indicating which of 24 chromosomes (chr1–22, X, Y) carried a mosaic alteration in each of the 38 MRD-evaluable patients. No significant associations with MRD status were identified: the AUC ranged from 0.54 to 0.63, and the two approaches retained different features. With only 13 MRD-positive events available for up to 24 candidate predictors, this result is expected rather than informative. The full analysis is reported in Supplementary Methods S1 and Table S3 for transparency.

We separately examined patients with ETP (n = 9) and near-ETP (n = 5) immunophenotypes. ETP phenotype was present in 3/13 MRD-positive (23.1%) and 6/25 MRD-negative patients (24.0%; *p* = 1.00). Among the four evaluable near-ETP patients, three were MRD-positive (*p* = 0.11, Fisher’s exact test); a fifth near-ETP patient died before day +70.

The two subtypes also showed different patterns of alteration (Supplementary Figure S3): chromosome 4 gains were seen in three ETP patients, whereas losses predominated among near-ETP—a 5q deletion in three patients and large-scale chromosome 16 and 17 events. Two near-ETP patients had concurrent 17p loss and 17q gain, a copy-number pattern compatible with but not diagnostic of isochromosome 17q; orthogonal confirmation by FISH or optical genome mapping was not performed, and conventional cytogenetics did not detect this rearrangement (Figure 4). Deletions at 9p occurred in both subtypes.

Finally, we restricted the analysis to the 57 cancer genes in our oncopanel (Figure 5, Supplementary Table S2). *CDKN2A* and *JAK2*, both located on 9p, were the two most frequently altered loci overall, irrespective of MRD status, although the type of event differed. For *CDKN2A*, mosaic deletion occurred in 3/13 MRD-positive and 2/25 MRD-negative patients, while cnLOH was confined to the MRD-negative group (5/25); for *JAK2*, deletion occurred in 3/13 and 2/25, and cnLOH occurred in 1/13 and 6/25. The two genes lie roughly 16 Mb apart, and in most cases, their co-alteration reflected a single extended 9p event rather than two independent lesions.

Beyond this shared locus, gene-level losses were more frequent in the MRD-positive group with two patients, showing concurrent losses spanning *NPM1*, *CSF1R*, and *TSC2*. MRD-negative patients more commonly showed isolated gains and a lower overall event density; one MRD-negative patient had a mosaic gain of nearly the entire chromosome 7, covering all panel genes on this chromosome.

## 3. Discussion

### 3.1. Summary of the Principal Findings

We examined whether the genomic changes detectable by CMA at diagnosis relate to early treatment response, measured as MRD positivity at day +70—the threshold that separates 35% from 79% 3-year relapse-free survival in our protocol and that drives referral to allogeneic transplantation [5]. Four observations emerged.

First, mosaic alterations were present in almost all patients (13/13 MRD-positive; 24/25 MRD-negative), so the groups differed in the amount of genomic change rather than in its presence. Second, burden measures such as complex CMA profiles, abnormal karyotype and oncopanel losses were numerically higher in MRD-positive disease, but none of these differences reached statistical significance. Third, the two mechanisms of 9p allelic loss were separable: biallelic 9p21.3 deletion occurred in both groups, whereas cnLOH without an underlying deletion was concentrated in MRD-negative patients, again without statistical significance. Overall, cnLOH burden did not differ between groups, unlike copy-number burden, indicating that the two classes of events are not interchangeable. Fourth, among the four evaluable near-ETP patients, three were MRD-positive, and this subgroup showed 5q deletion together with concurrent 17p loss and 17q gain.

With only 13 and 25 patients per group, the study is underpowered for formal inference. The observations above describe this cohort and should not be read as established associations.

### 3.2. Burden Rather than Presence, and Two Classes of Events

Nearly every T-ALL genome carries some alteration at diagnosis [1,3,6,7], so a simple “abnormal versus normal” readout adds little. CMA detected alterations in more patients than karyotyping, consistent with the known tendency of karyotyping to underestimate genomic complexity in T-ALL [2,8,9] and with our earlier finding that karyotype at diagnosis does not predict survival in this cohort [5]. The two methods are complementary rather than ranked: in one patient, CMA showed no copy-number alterations, only a region of cnLOH, whereas karyotyping showed a t(3;9) in a single metaphase; rearrangements of this kind leave copy number unchanged and are invisible to arrays.

The clearest structural observation is that copy-number burden and cnLOH burden did not track together: copy-number burden was numerically higher in MRD-positive patients, although without statistical significance, whereas cnLOH burden was almost identical between groups. This is biologically coherent—gains and losses arise from unbalanced structural events, while cnLOH mostly arises from mitotic recombination and leaves gene dosage unchanged [10,11]. We suggest both classes be scored separately rather than merged into a single “instability” index, as has been proposed for myeloid neoplasms [10]. This also has a practical dimension: cnLOH is invisible to karyotyping and FISH, and SNP-array platforms such as CMA remains the only routine method that captures both classes from the same sample [8,9,10,12].

### 3.3. Mechanisms of Allelic Loss at 9p21.3: Deletion Versus cnLOH

The most consistent pattern in our data concerns 9p. The *CDKN2A/CDKN2B* locus is the most frequently altered region in T-ALL [1,3,4], and we previously documented biallelic loss of this locus together with 7q34 (*TRB*) in adult Ph-negative T-ALL treated on the same protocols [13]. Inactivation of 9p21.3 can occur through a focal biallelic deletion, a monoallelic deletion, or through cnLOH duplicating an already defective allele—mechanisms usually lumped together as “9p alteration”. Our data suggest they should not be.

Among the seven patients with 9p cnLOH, three also carried a nested biallelic deletion—consistent with mitotic recombination duplicating a chromosome that already carried a deleted allele—while four kept two intact copies. Biallelic 9p21.3 loss itself, regardless of mechanism, showed no clear association with MRD status (nine patients overall, numerically more frequent in the MRD-negative group). A descriptive difference was observed for cnLOH without an underlying deletion, which occurred predominantly in MRD-negative patients, although this did not reach statistical significance. Notably, nearly all patients with biallelic 9p21.3 loss (8/9) had a non-ETP immunophenotype, matching the known enrichment of 9p deletions in more mature (cortical) disease [8] and confounding any potential independent association with treatment response in this cohort.

Prior studies support treating deletion and cnLOH separately: pediatric ALL cases with *CDKN2A* LOH clustered with favorable early response, while biallelic deletion marked more aggressive, MRD-persistent disease [14]. Phylogenetic reconstruction placed 9p events, including deletion and LOH, at the root of leukemic clone, ahead of *NOTCH1* mutations [15]. More broadly, cnLOH is typical and non-random across adult ALL genomes [16] and carries measurable long-term prognostic significance in childhood ALL [17], while targeting LOH itself is being explored as a therapeutic strategy [18]. If cnLOH marks an earlier, simpler clonal architecture and deletions arise later in more complex genomes, this would offer one explanation for the pattern we observed, but we cannot test this directly. Our data are single-timepoint with no clonal reconstruction, the comparator series differ in population (pediatric, including T-lymphoblastic lymphoma, whose genomic landscape is related but not identical [19,20,21]) and in age distribution [15], and our 3 Mb LOH threshold is more permissive than that used by Styka et al. [14].

### 3.4. Oncopanel Annotation

Restricting the analysis to the 57-gene target panel showed the same distribution as the genome-wide findings. Gene-level losses were more frequent in the MRD-positive group (3.0 versus 0.6 events per patient), whereas cnLOH was more frequent in the MRD-negative group (1.5 versus 0.8 events per patient). None of these differences reached statistical significance.

Two methodological caveats apply. First, microarray analysis detects structural dosage changes rather than functional status, leaving key sequence-level alterations such as activating *NOTCH1* or *FBXW7* mutations undetected [22,23,24]. Second, a single large structural event necessarily credits every gene within its boundaries, potentially inflating gene-level counts—a limitation inherent to array-based methods [8,25].

Given the limited sample size (n = 38; 13 MRD-positive events), multivariate predictive modeling was not retained in the primary analysis, as the low event-to-variable ratio makes formal predictive inference inappropriate. All structural alterations reported here are therefore treated as descriptive genomic features rather than candidate predictors of MRD response.

### 3.5. Immunophenotype at Presentation: ETP and Near-ETP

MRD-negative patients presented with higher white-cell counts, higher LDH and higher blast percentages, all classically “adverse” features. We interpret this as reflecting the subtype mix rather than a true reversal of prognosis: MRD-positive patients were enriched for immature (ETP/near-ETP) phenotypes, which typically present with lower counts [26,27], while the cortical (T-III) subtype, more common among MRD-negative patients, presents with high tumor burden but responds well to intensive therapy [4,8,28]. Presenting burden and treatment response appear to be separate axes, and it is the response that carries prognostic weight [21,29,30].

ETP status alone did not identify poor early responders here. This differs from the original description of ETP-ALL as very high risk [27,31,32] but matches more recent evidence that the effect is smaller under pediatric-inspired protocols like ours [25,33,34]. Among the four evaluable near-ETP patients, three were MRD-positive, and their profiles were dominated by losses (5q deletion, large chromosome 16 and 17 events), whereas the three ETP patients with chromosome 4 involvement showed gains. Two near-ETP patients had concurrent 17p loss and 17q gain, a copy-number configuration compatible with, but not diagnostic of, isochromosome 17q, which karyotyping did not detect [8,9,12]; confirmation by FISH or optical genome mapping was not performed. With three to five patients per subgroup, these observations are descriptive rather than statistically robust [26].

### 3.6. Limitations and Implications

This is a small, single-center, single-protocol, descriptive study: 38 evaluable patients, no correction for multiple testing, no validation cohort, and the near-ETP observation resting on four patients. We analyzed only mosaic events, which better reflect an admixed marrow sample but may miss some clonal lesions; without a matched remission sample, somatic origin cannot be formally established for any event. We used a 3 Mb cnLOH threshold that limits comparison with other studies [14], and CMA cannot detect balanced rearrangements or point mutations that are independently prognostic in T-ALL [8,12,22,23].

The cohort size places a hard limit on what could be detected. With 13 MRD-positive and 25 MRD-negative patients, Fisher’s exact test at a two-sided α of 0.05 reaches 80% power only for differences corresponding to odds ratios of approximately 8–10—for example, 20% versus 68%. For the differences actually observed, post-hoc power was 0.09 for 9p cnLOH and 0.33 for complex profiles. Confirming the 9p cnLOH observation at 80% power would require roughly 190 patients in the same group proportions, and confirming the complex-profile observation would require roughly 120 patients. The absence of statistical significance here is therefore uninformative about whether these associations exist, and no negative conclusion should be drawn from it either.

Two implications follow from the descriptive data themselves, independently of any association with MRD. First, 9p events are better reported by mechanism—deletion versus cnLOH—than pooled, which requires SNP-array platforms capable of resolving both [9,10]. Second, near-ETP cases may warrant separate analysis from ETP, which current classifications do not yet do [35,36]; immature T-ALL subtypes are already a specific focus of functional precision-medicine trials in the relapsed/refractory setting [37], within a broader effort to develop targeted approaches in T-ALL [38]. Day +70 MRD remains the strongest available measure of early treatment response [5,29,30,39,40]; the question this study addressed was whether a baseline genomic feature could anticipate that measure before it becomes available, and in this cohort, none did.

## 4. Materials and Methods

### 4.1. Patients and Treatment

Thirty-nine adults with Ph-negative T-ALL formed the study group. All were treated at the National Medical Research Center for Hematology between 2017 and 2026 under the RALL-2016m protocol [5] and had diagnostic tumor DNA available for retrospective analysis. RALL-2016m was derived from the earlier RALL-2009 and RALL-2016 regimens and retained their central design principle: moderate-intensity chemotherapy delivered without interruption. Assessment of MRD on day +70 was built into the protocol and marked the end of induction.

### 4.2. Cytogenetic and FISH Studies

Diagnostic bone marrow was submitted for immunophenotyping together with cytogenetic and molecular investigation. Metaphase preparations were examined after GTG banding, with 20 metaphases analyzed where available; analyzable metaphases were obtained in 36 of 39 patients. *BCR::ABL1* rearrangement was ruled out by FISH in all cases (BCR/ABL (DF) Gene Fusion Probe Detection Kit; Wuhan HealthCare Biotechnology, Wuhan, China). Depending on the karyotype result, further probes interrogated *KMT2A*/11q23 (XL MLL plus Break Apart Probe; MetaSystems, Altlussheim, Germany), *TRA*/*TRD*/14q11 and *IGH*/14q32 (TCRA/D and IGH Break Apart Probes; MetaSystems), *BCL11B*/14q32 (BCL11B Break Apart FISH Probe; Empire Genomics, New York, NY, USA), *TP53*/17p13 (P53/CEP17 Deletion Probe; Wuhan HealthCare Biotechnology), and the *PDGFRA* and *PDGFRB* loci (corresponding Gene Break Apart Probe Detection Kits; Wuhan HealthCare Biotechnology). Karyotypes and FISH findings were reported following ISCN 2020 [41].

### 4.3. MRD Monitoring

Once T-ALL was confirmed, the leukemia-associated immunophenotype was characterized at onset so that residual disease could be tracked in follow-up samples. Detection relied on the different-from-normal approach, in which immunophenotypically immature T cells are identified against the background of normal marrow. Instrumentation changed during the study period: samples processed up to 2020 were acquired on a BD FACSCanto II (BD Biosciences, San Jose, CA, USA) with a six-color panel split across two tubes (CD99, CD7, cytoplasmic and surface CD3, CD5, CD45, CD8), whereas from 2021 onward a single-tube ten-color panel was run on a Beckman Coulter CytoFLEX (Beckman Coulter, Brea, CA, USA) (CD99, CD7, CD34, CD56, cytoplasmic and surface CD3, CD4, CD5, CD45, CD8).

Between 500,000 and 1,000,000 events were acquired per sample, giving an analytical sensitivity of 10^−5^. Samples were scored as MRD-positive when the residual population exceeded 0.01% of nucleated cells; values from 0 to 0.01% were scored as MRD-negative. The two panels were compared during the transition through an internal validation program using bone marrow from healthy donors and from individual patients, which showed the two approaches to be comparable. Irrespective of the panel, MRD was measured in bone marrow at the day +70 end-of-induction timepoint.

### 4.4. Chromosomal Microarray Analysis

Genomic DNA extracted from pre-treatment bone marrow served as input for CMA. Samples were required to yield 100–200 ng of DNA with an A260/A280 ratio of at least 1.8; commercial male reference DNA (Thermo Fisher Scientific, Waltham, MA, USA) at matching concentration was processed in parallel. Hybridization was carried out on CytoScan HT-CMA 96F SNP-oligonucleotide arrays following the manufacturer’s instructions (Thermo Fisher Scientific, Santa Clara, CA, USA) at the Genomed Laboratory of Molecular Pathology (Moscow, Russia). Array data were reviewed in Multi Sample Viewer (v1.1.0.11) and Chromosome Analysis Suite (ChAS v4.3.0.71). Regions of LOH were called only when they reached 3 Mb or more, a threshold adopted from Gonzales et al. [42]. Bone marrow specimens contain admixed non-leukemic cells, so only mosaic events were carried forward into the analyses reported here.

### 4.5. Restriction of the CMA Output to a Cancer Gene Set

To focus interpretation on loci of established oncogenic relevance, we defined a target gene set in advance rather than surveying the genome without restriction. The set reproduces the content of the commercial xGen 57G Pan-Cancer Amplicon Panel (Integrated DNA Technologies, Coralville, IA, USA), which covers 57 genes recurrently involved in hematological and solid malignancies: *ABL1*, *AKT1*, *ALK*, *APC*, *ATM*, *BRAF*, *CDH1*, *CDKN2A*, *CSF1R*, *CTNNB1*, *DDR2*, *DNMT3A*, *EGFR*, *ERBB2*, *ERBB4*, *EZH2*, *FBXW7*, *FGFR1*, *FGFR2*, *FGFR3*, *FLT3*, *FOXL2*, *GNA11*, *GNAQ*, *GNAS*, *HNF1A*, *HRAS*, *IDH1*, *IDH2*, *JAK2*, *JAK3*, *KDR*, *KIT*, *KRAS*, *MAP2K1*, *MET*, *MLH1*, *MPL*, *MSH6*, *NOTCH1*, *NPM1*, *NRAS*, *PDGFRA*, *PIK3CA*, *PTEN*, *PTPN11*, *RB1*, *RET*, *SMAD4*, *SMARCB1*, *SMO*, *SRC*, *STK11*, *TP53*, *TSC1*, *TSC2*, and *VHL*. Copy-number alterations and cnLOH segments called by CMA were retained for this part of the analysis only where their genomic coordinates strictly overlapped one or more of these loci; all other events were set aside. The resulting gene-level landscapes were then compared between the MRD-positive and MRD-negative cohorts.

### 4.6. Statistical Analysis

MRD status at day +70 served as the endpoint throughout. Categorical data were summarized as frequencies and compared between groups by Fisher’s exact test; continuous variables were summarized as means and ranges and compared by Student’s t-test. All tests were two-sided, with α set at 0.05. No correction for multiple testing was applied, and all *p*-values are reported descriptively rather than as a basis for inference.

Statistical power was evaluated post hoc for the group sizes obtained (13 MRD-positive, 25 MRD-negative), using the exact distribution of Fisher’s test to determine both the minimum difference detectable at 80% power and the power attained for the differences actually observed.

Exploratory chromosome-level modeling was performed on a binary matrix recording the presence of any mosaic alteration on each of the 24 chromosomes (chr1–22, X, Y) across the 38 MRD-evaluable patients, using LASSO-penalized logistic regression (glmnet) and random forest (ranger), with AUC calculated using pROC. Because the cohort provides approximately 0.5 outcome events per candidate predictor, well below the recommended minimum of ten, these analyses are reported in Supplementary Methods S1 and Table S3 as a negative result and are not part of the primary analysis; no model is proposed for clinical use and no inference is drawn from the fitted coefficients.

All computations were performed in R 4.6.1 (R Foundation for Statistical Computing, Vienna, Austria) and SAS 9.4 (SAS Institute Inc., Cary, NC, USA); the R packages glmnet (https://cran.r-project.org/package=glmnet, accessed on 20 October 2026), ranger (https://cran.r-project.org/package=ranger, accessed on 20 October 2026) and pROC (https://cran.r-project.org/package=pROC, accessed on 20 October 2026) were used.

## 5. Conclusions

The cohort of 38 MRD-evaluable patients is too small to support formal inference, and no association with MRD status reached statistical significance.

Two observations do not depend on such an association. Copy-number and copy-neutral events behaved as separate phenomena in this cohort and are therefore best scored separately. At 9p21.3, this distinction was visible directly: cnLOH without an underlying deletion occurred predominantly in MRD-negative patients, whereas biallelic loss occurred in both groups—a difference that disappears when 9p events are pooled as a single category.

Descriptively, genomic burden—complex CMA profiles, abnormal karyotype, and oncopanel losses—was numerically higher in MRD-positive patients, and among the four evaluable near-ETP patients, three were MRD-positive, with 5q deletion and concurrent 17p loss and 17q gain. These counts describe the present cohort and require confirmation in larger, preferably multicenter series, in which 9p alterations are reported by mechanism, near-ETP is analyzed separately from ETP, and array-based profiling is complemented by sequencing.

## Figures and Tables

**Figure 1:**
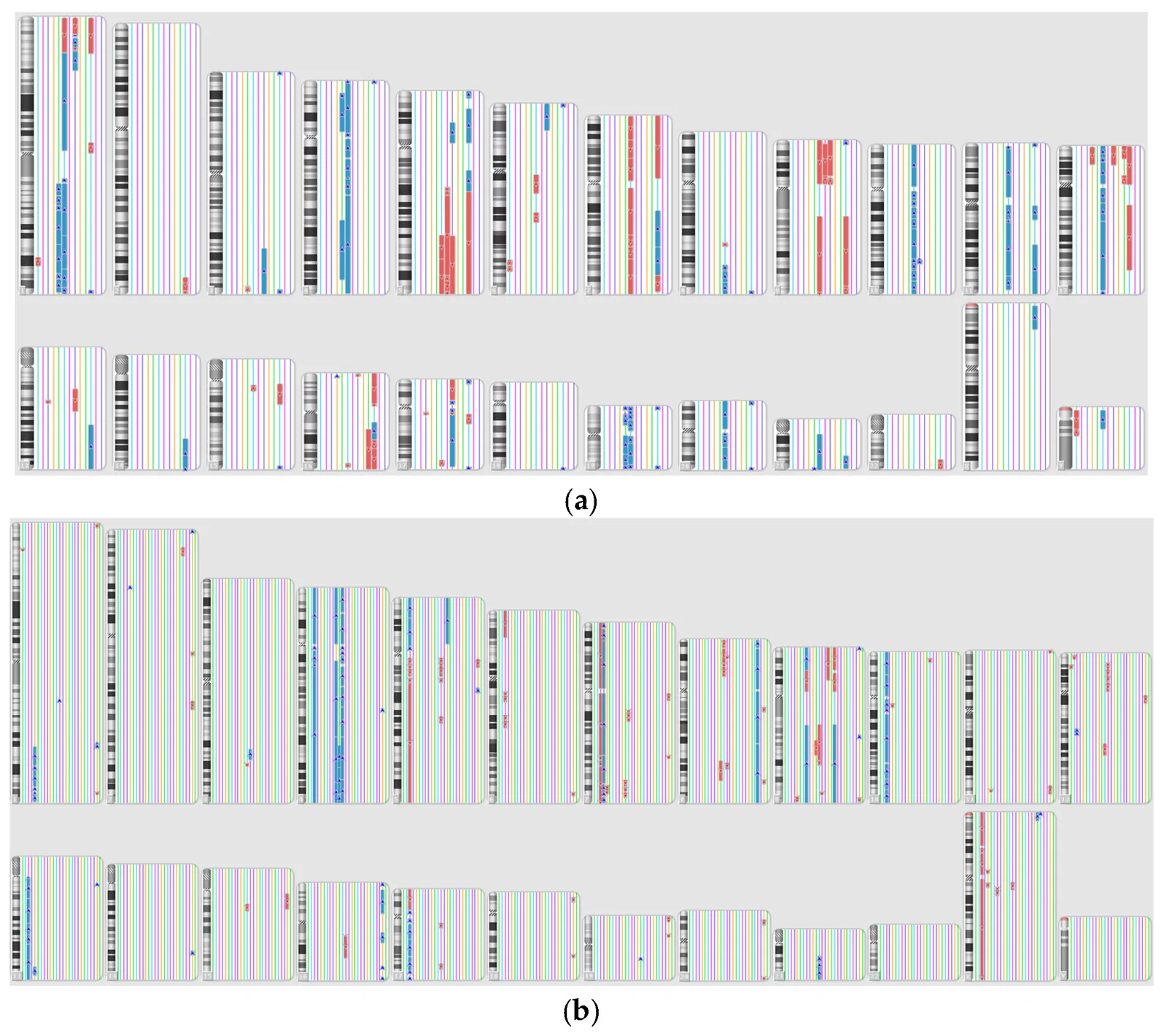
Chromosomal copy-number alterations in MRD-positive (**a**) and MRD-negative (**b**) T-ALL patients. Red bars indicate losses; blue bars indicate gains. Complex profiles (≥3 affected chromosomes) were more frequent in the MRD-positive group (46.2% vs. 20.0%).

**Figure 2:**
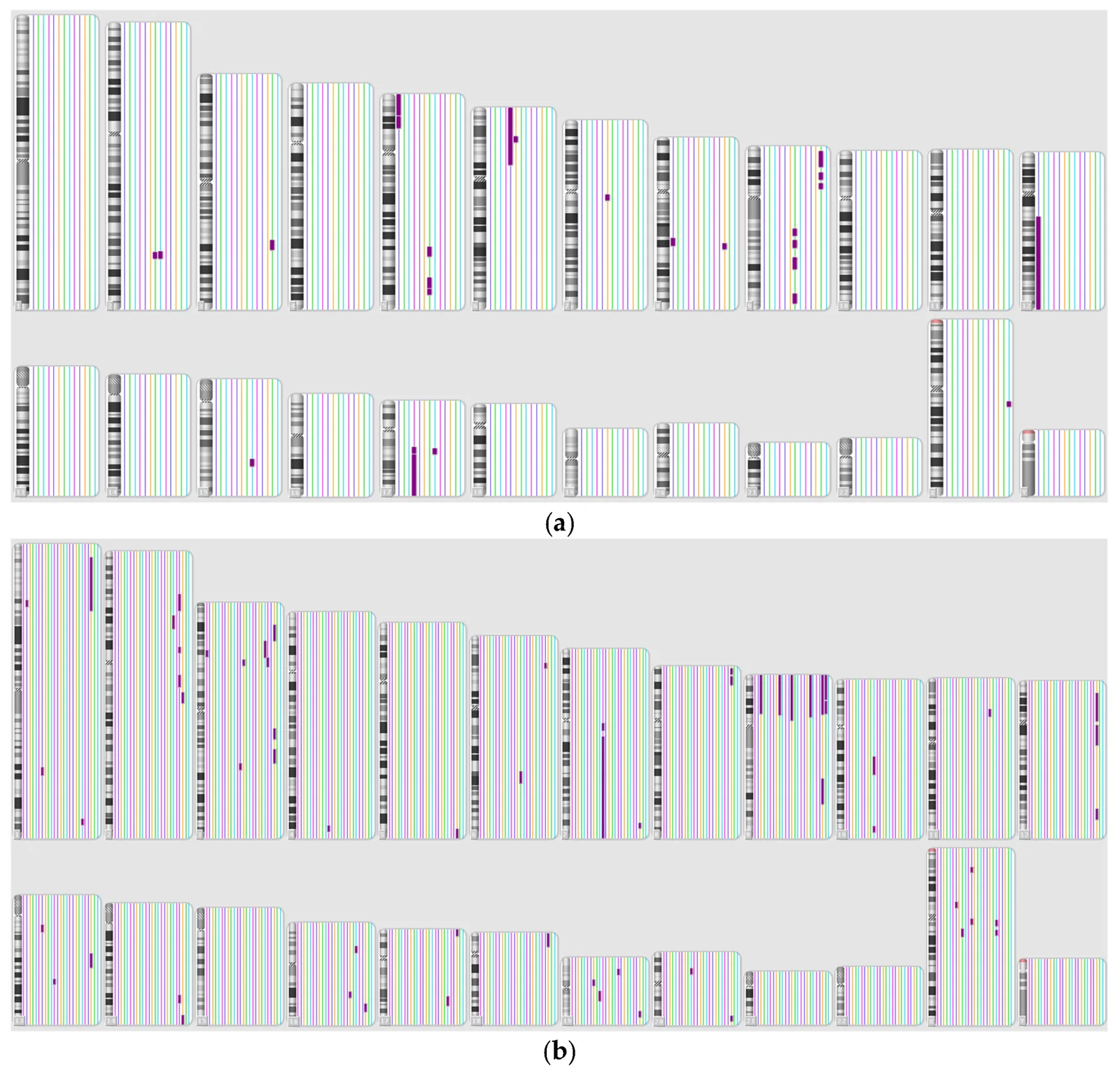
Copy-neutral loss of heterozygosity (cnLOH) in MRD-positive (n = 13) (**a**) and MRD-negative (n = 25) (**b**) T-ALL patients. Purple bars mark cnLOH regions of 5 Mb or more. All analyses included cnLOH regions of 3 Mb or more (Section 4.4).

**Figure 3:**
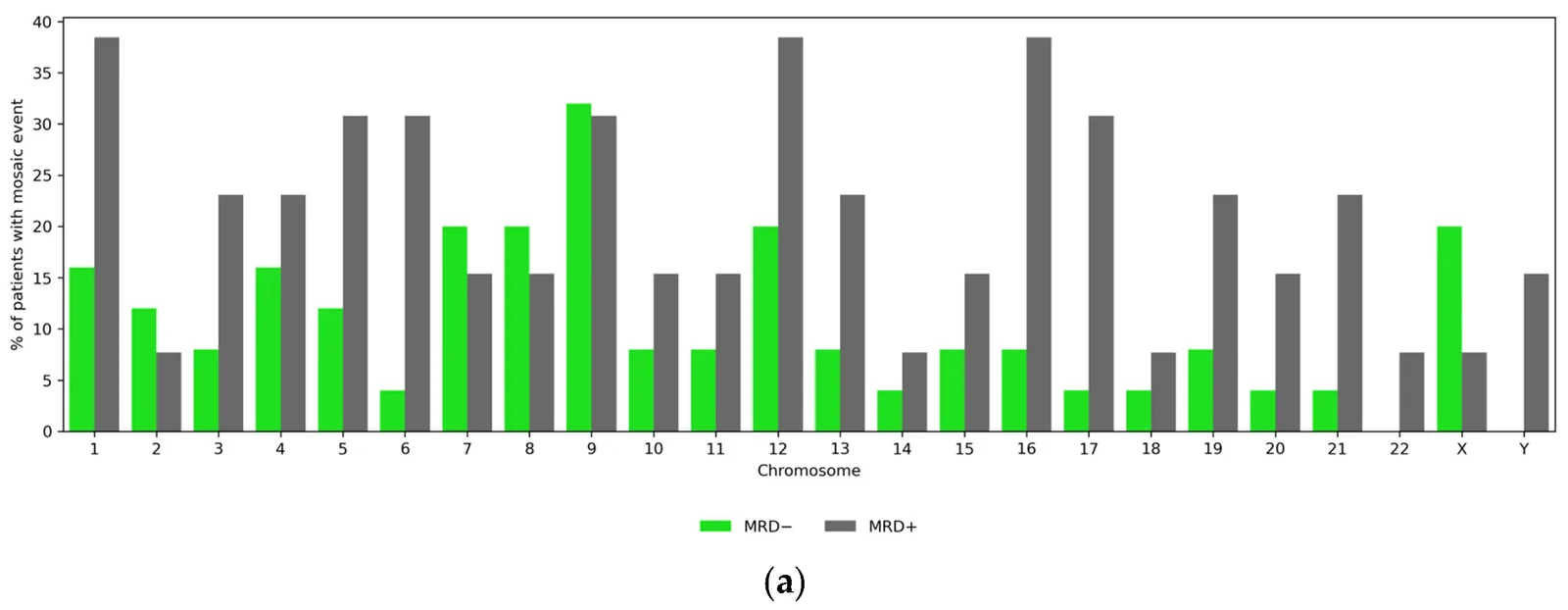

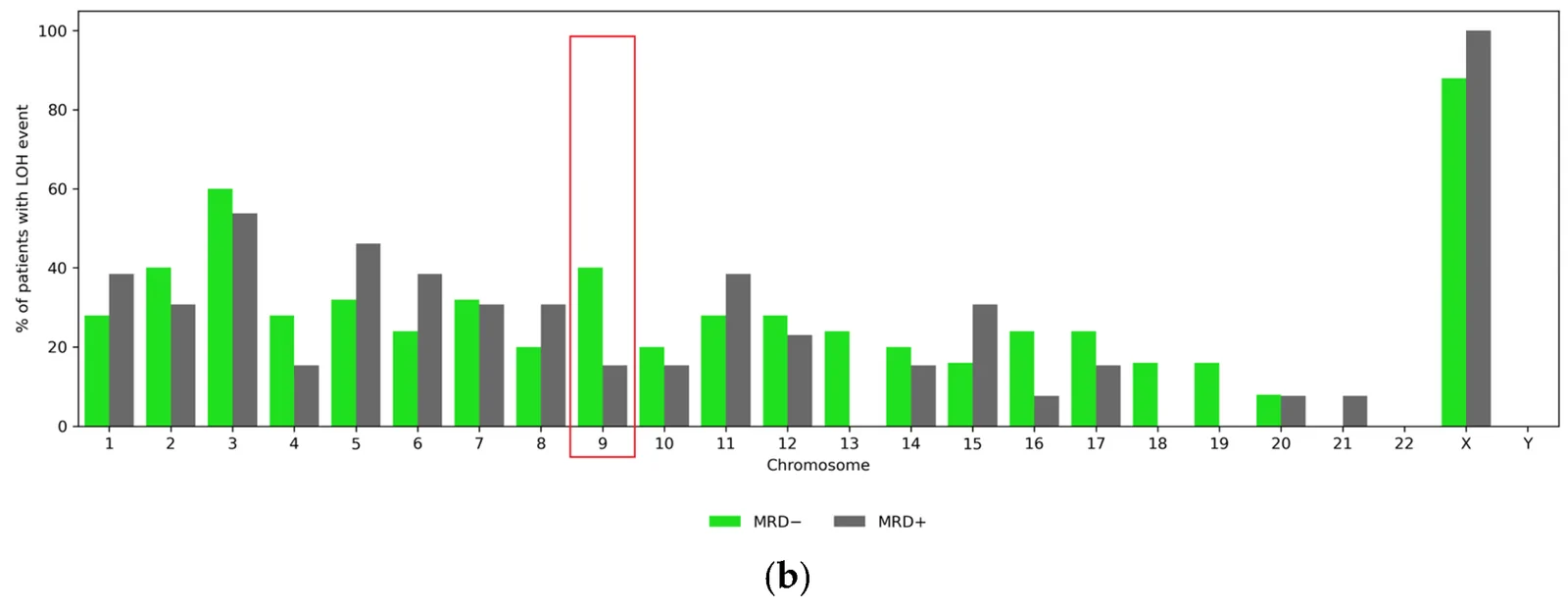
Genomic distribution of copy-number alterations (**a**) and cnLOH events (**b**) by MRD status. Bars show the percentage of patients with each aberration per whole chromosome (green bars indicate MRD-negative patients, n = 25; grey bars indicate MRD-positive patients, n = 13). The red frame in (**b**) marks chromosome 9, where cnLOH was more frequent in MRD-negative patients.

**Figure 4:**
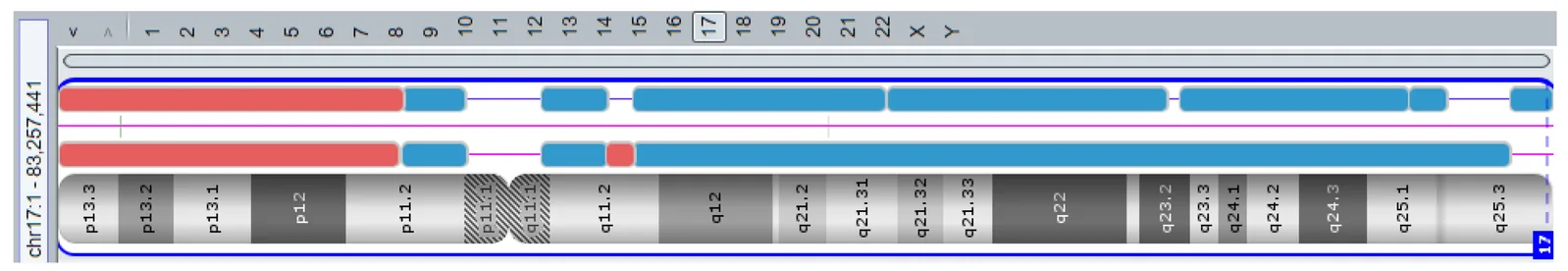
Combination of 17p loss and 17q gain, and detailed view of chromosome 17 in two near-ETP cases. Red bars indicate losses; blue bars indicate gains.

**Figure 5:**
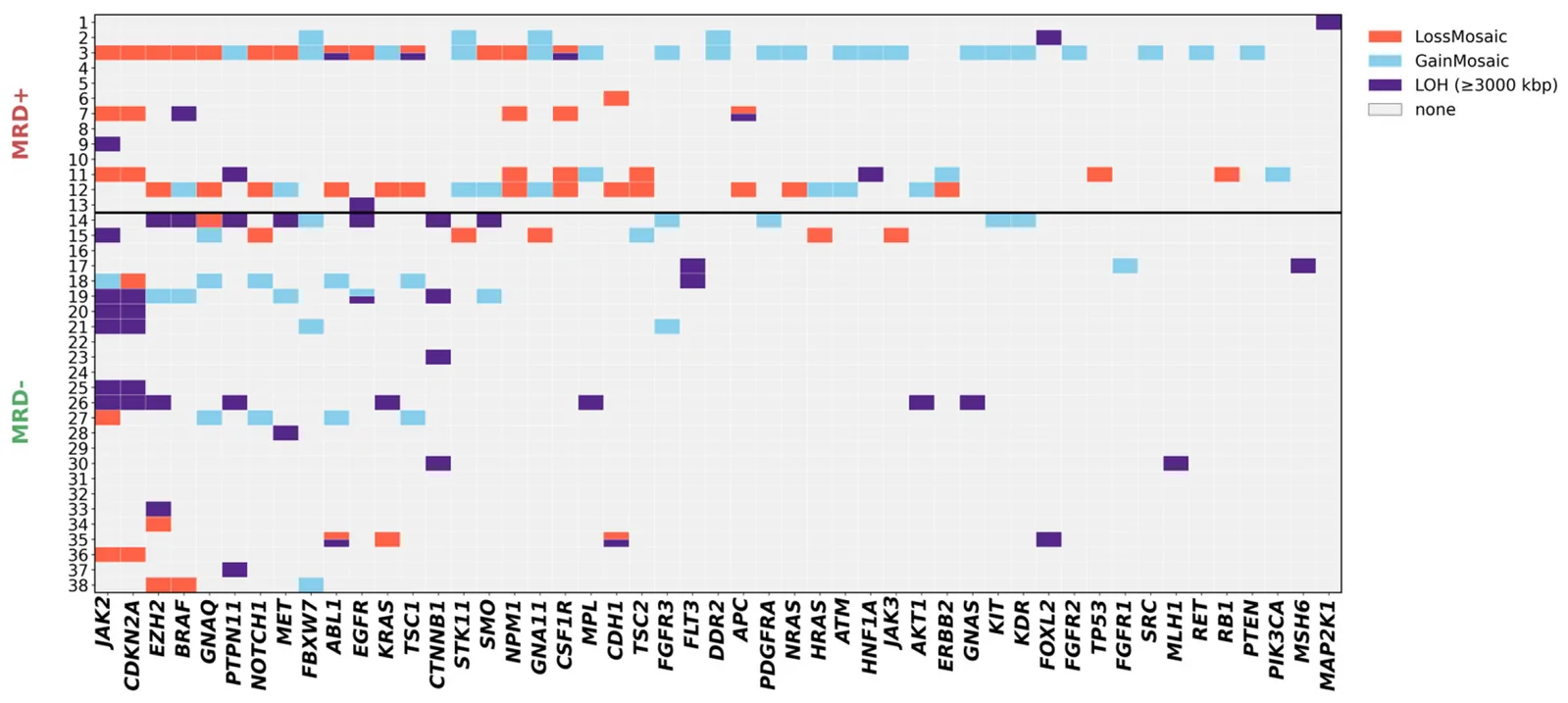
Oncoprint of copy-number alterations and cnLOH in the 57-gene panel. Rows are patients, and columns are genes with at least one detected event; patients are stratified by MRD status (upper: MRD-positive; lower: MRD-negative). Red bars indicate losses; blue bars indicate gains; purple bars indicate cnLOH. The black horizontal line separates MRD-positive (upper) and MRD-negative (lower) patients.

**Table 1:** Main characteristics of patients according to MRD status.

Parameters	MRD+ (n = 13)	MRD− (n = 25)	Early Death (n = 1)	Total (n = 39)
Male/female ratio	9:4	19:6	0:1	28:11
Age, years, mean (range)	36 (23–55)	33 (21–53)	30	34 (21–55)
Leukocytes, 10^9^/L, mean (range)	55.83 (2.44–445)	108.92 (0.95–355.28)	116	90.46 (0.95–445)
LDH, U/L, mean (range)	764 (154–3601)	2725.37 (333–7633.2)	2389	1987.28 (154–7633.2)
Blast cells in peripheral blood, %, mean (range)	33.92 (0–83)	59.52 (0–97)	84	51.21 (0–97)
Blast cells in the bone marrow, %, mean (range)	57.39 (5.2–92)	82.83 (30.5–100)	84	74.38 (5.2–100)
Immunophenotype, EGIL, WHO				
T-I	3	3	-	6
T-II	7	7	1	15
T-III	2	12	-	14
T-IV	-	1	-	1
MPAL (T-myelo)	1	2	-	3
ETP status				
ETP	3	6	-	9
near-ETP	3	1	1	5
non-ETP	7	18	-	25
Standard cytogenetics	13	22	1	36
+mitosis	13	22	1	36
−mitosis	-	3	-	3
Karyotype				
Normal	5	11	-	16
Abnormal	8	11	1	20
CNS leukemia	4	4	-	8
Extramedullary disease	10	16	1	27

CNS, central nervous system; EGIL, European Group for the Immunological Characterization of Leukemias; ETP, early T-cell precursor; LDH, lactate dehydrogenase; MPAL, mixed-phenotype acute leukemia; MRD, measurable (minimal) residual disease; MRD+, MRD-positive; MRD−, MRD-negative; n, number of patients; near-ETP, near early T-cell precursor; T-I to T-IV, EGIL immunological subtypes of T-lineage acute lymphoblastic leukemia (T-I, pro-T; T-II, pre-T; T-III, cortical T; T-IV, mature T); WHO, World Health Organization.

## Data Availability

Data supporting the findings are contained within the article and the Supplementary Materials. Further data may be made available by the corresponding author subject to the authorization procedures required by Russian Federation legislation on the cross-border transfer of human genetic data (Government Decree No. 750 of 15 June 2026).

## Supplementary Materials

The following supporting information can be downloaded at https://www.mdpi.com/article/10.3390/ijms27209348/s1.

## Abbreviations

The following abbreviations are used in this manuscript:

MRD	measurable (minimal) residual disease
T-ALL	T-cell acute lymphoblastic leukemia
CMA	chromosomal microarray analysis
cnLOH	copy-neutral loss of heterozygosity
AUC	area under the curve
ETP	early T-cell precursor
ALL	acute lymphoblastic leukemia
DNA	deoxyribonucleic acid
CNA	copy-number alteration
LDH	lactate dehydrogenase
EGIL	European Group for the Immunological Characterization of Leukemias
WHO	World Health Organization
MPAL	mixed-phenotype acute leukemia
CNS	central nervous system
FISH	fluorescence in situ hybridization
SNP	single-nucleotide polymorphism
LOH	loss of heterozygosity
Ph	Philadelphia chromosome
RALL	Russian Acute Lymphoblastic Leukemia (study protocol)
GTG	G-bands by trypsin using Giemsa
ISCN	International System for Human Cytogenomic Nomenclature
