# Supplementary material for: Copy-Number Alterations and Copy-Neutral Loss of Heterozygosity in Adult T-ALL: A Descriptive Study in Relation to Day +70 MRD
Source: Int J Mol Sci. 2026 Oct 21;27(20):9348. doi: 10.3390/ijms27209348 (PMC13705239; doi:10.3390/ijms27209348)
Supplement: Supplementary file 1 [file ijms-27-09348-s001.zip › Supplementary Figure S1.pdf]

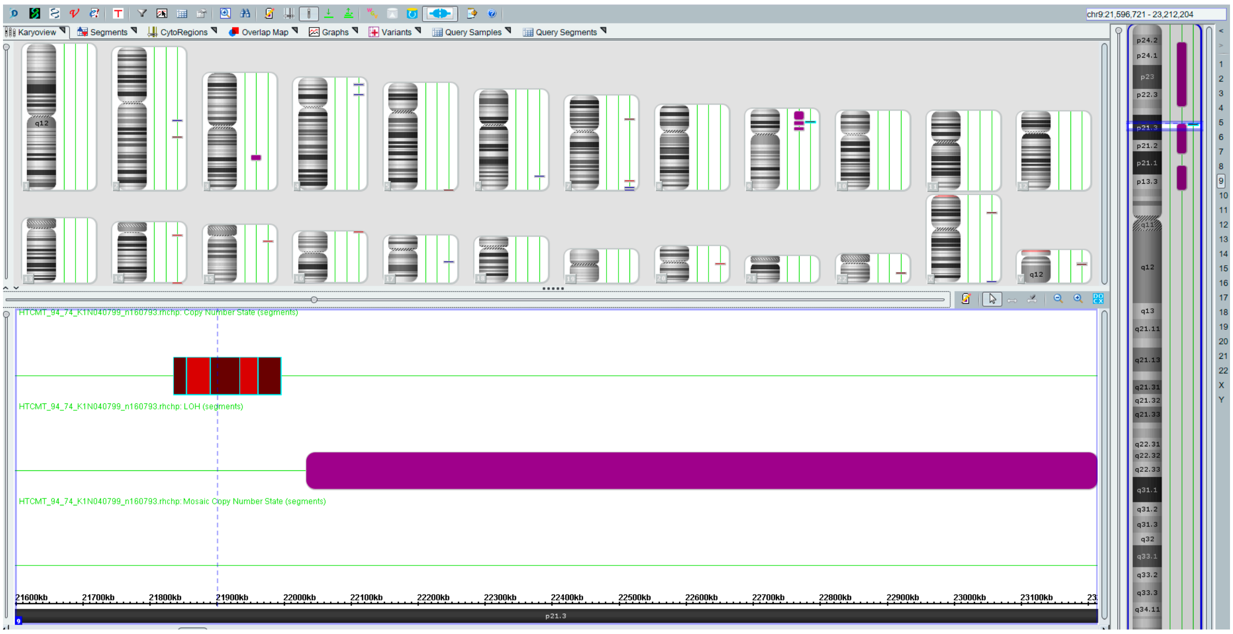

(**a**)

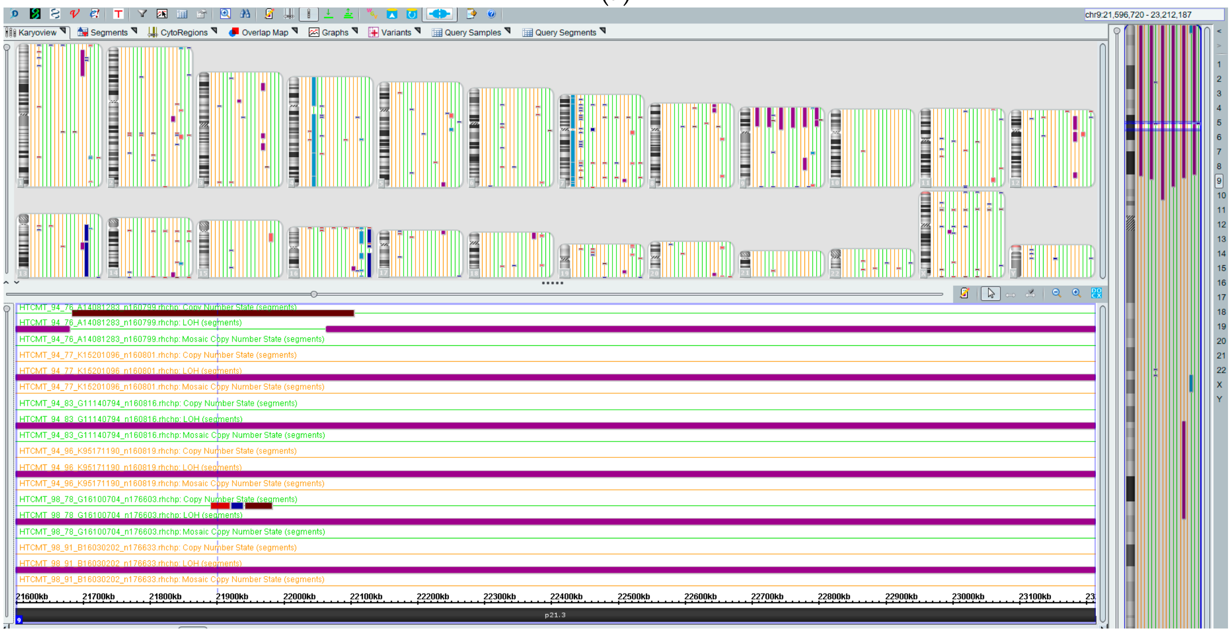

(**b**)

Figure S1. Biallelic deletion of 9p21.3 nested within a region of copy-neutral loss of heterozygosity. Detailed karyoview of chromosome 9 in the three patients in whom the cnLOH segment contained a focal biallelic deletion at 9p21.3: one MRD-positive patient (a) and two MRD-negative patients (b). *Red bars indicate losses; purple bars indicate cnLOH.*
