# Supplementary material for: Copy-Number Alterations and Copy-Neutral Loss of Heterozygosity in Adult T-ALL: A Descriptive Study in Relation to Day +70 MRD
Source: Int J Mol Sci. 2026 Oct 21;27(20):9348. doi: 10.3390/ijms27209348 (PMC13705239; doi:10.3390/ijms27209348)
Supplement: Supplementary file 1 [file ijms-27-09348-s001.zip › Supplementary Figure S2.pdf]

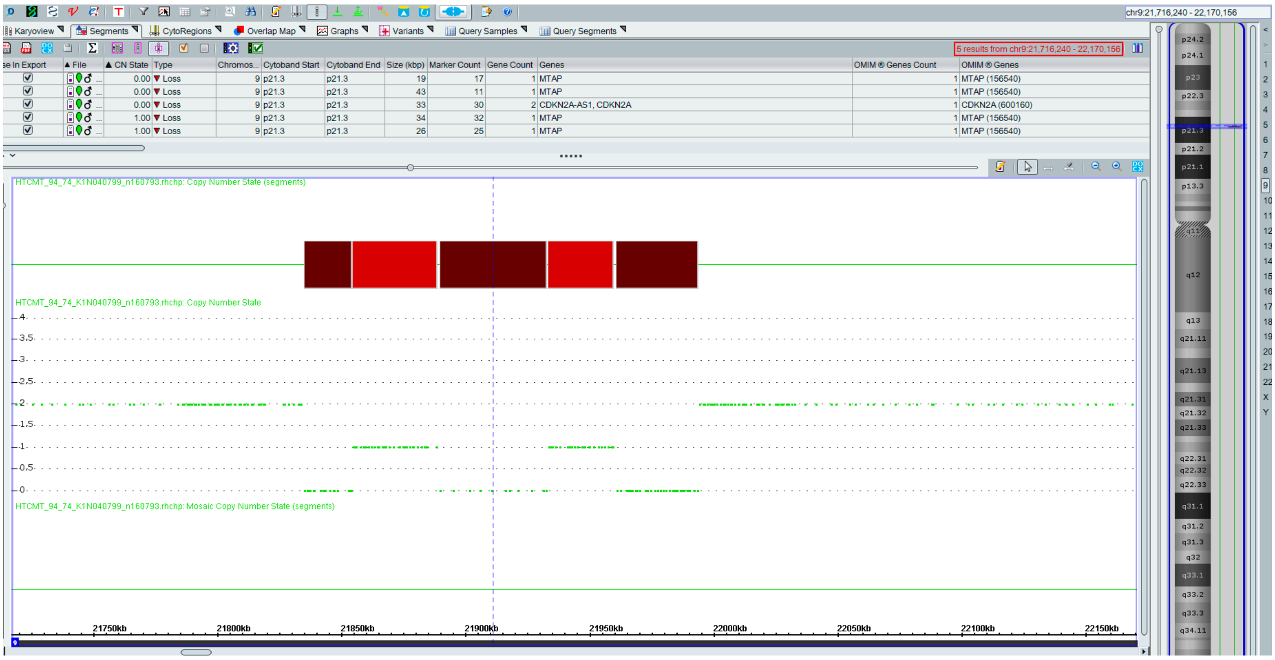

(**a**)

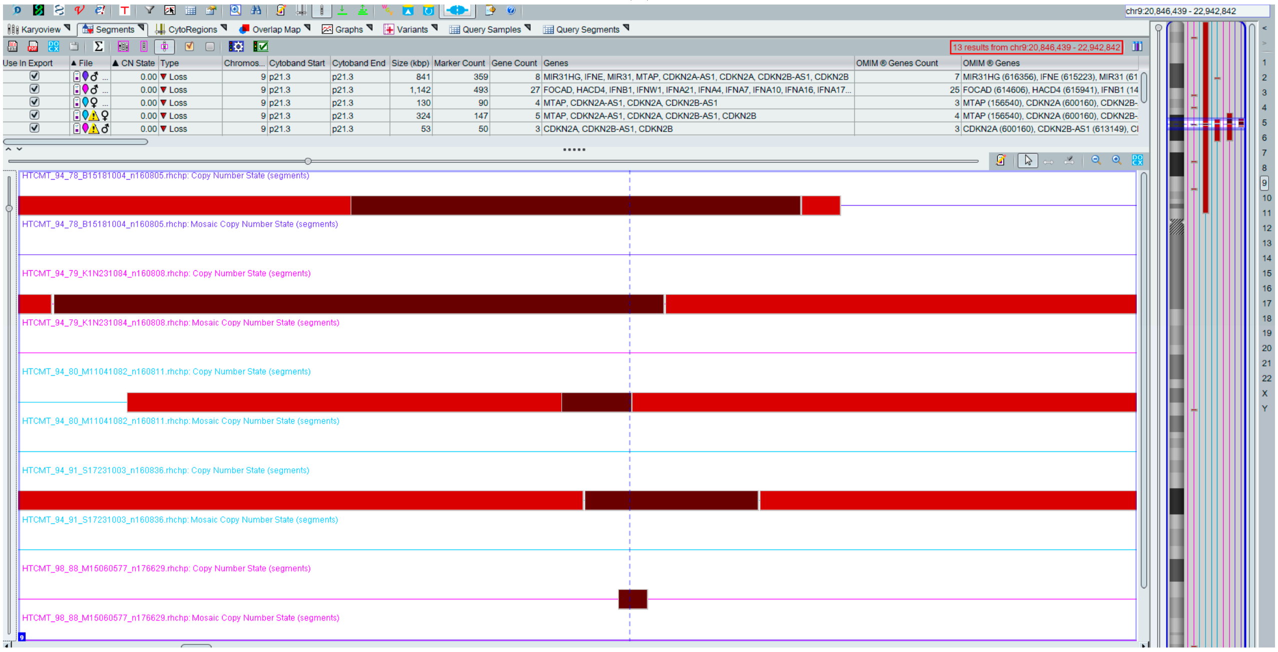

(**b**)

Figure S2. Biallelic deletion of 9p21.3 outside regions of copy-neutral loss of heterozygosity. Detailed view of chromosome 9 in the six patients with biallelic 9p21.3 deletion on a background of retained heterozygosity: one MRD-positive patient (a) and five MRD-negative patients (b). *Red bars indicate losses, deep red bars indicate biallelic losses.*
