# Supplementary material for: Copy-Number Alterations and Copy-Neutral Loss of Heterozygosity in Adult T-ALL: A Descriptive Study in Relation to Day +70 MRD
Source: Int J Mol Sci. 2026 Oct 21;27(20):9348. doi: 10.3390/ijms27209348 (PMC13705239; doi:10.3390/ijms27209348)
Supplement: Supplementary file 1 [file ijms-27-09348-s001.zip › Supplementary Figure S3.pdf]

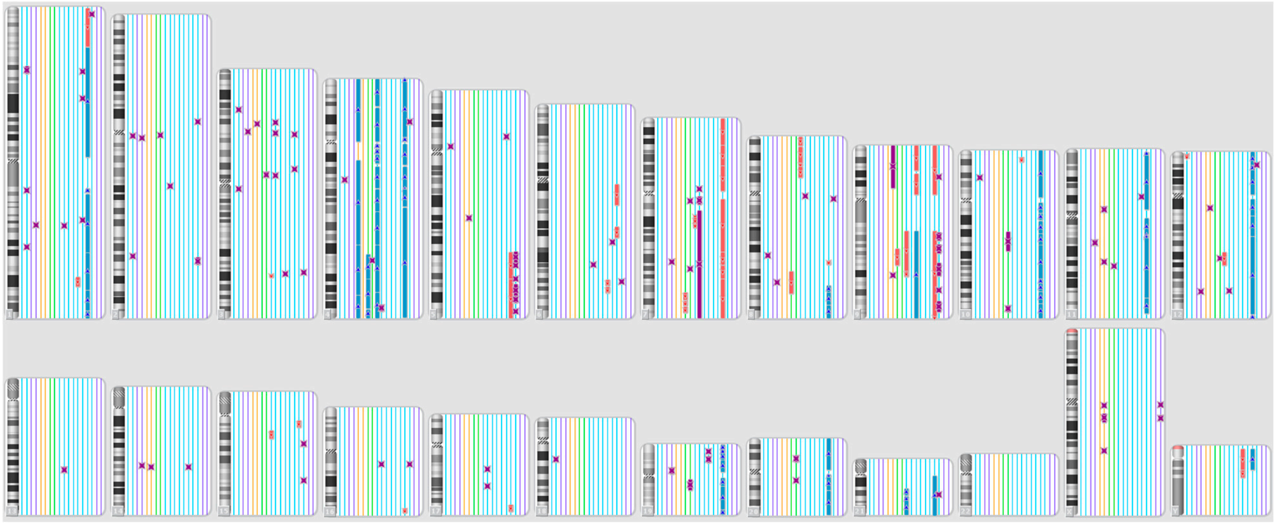

(a)

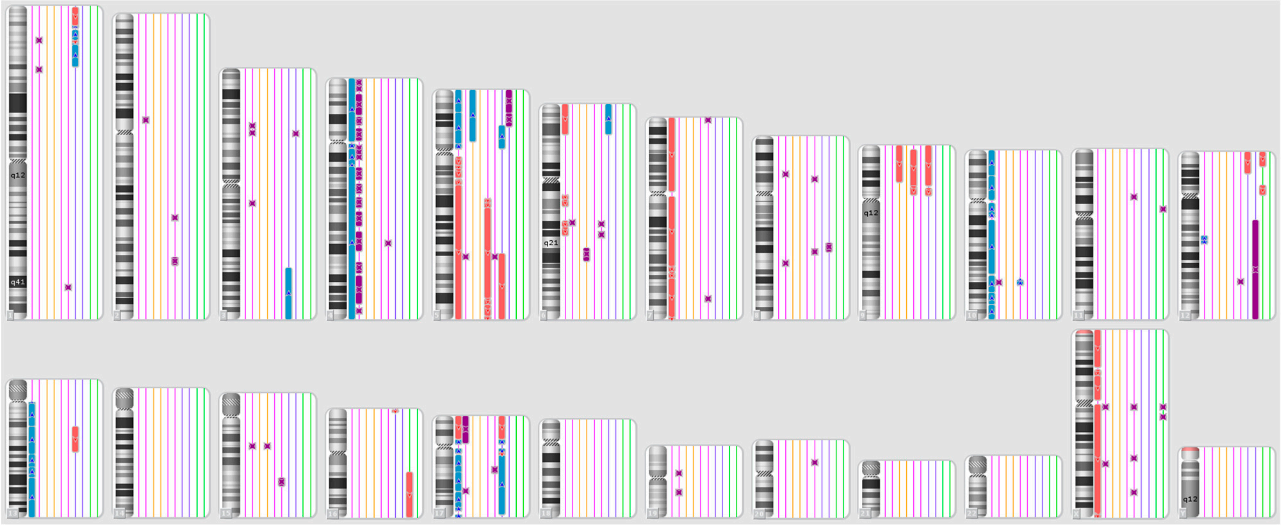

(b)

Figure S3. Composite molecular karyotypes of ETP (a) and near-ETP (b) T-ALL patients. *The ideograms summarize recurrent copy-number alterations identified by CMA in ETP (n=9) and near-ETP (n=5) patients. Red bars indicate losses; blue bars indicate gains.*
