# Supplementary material for: Copy-Number Alterations and Copy-Neutral Loss of Heterozygosity in Adult T-ALL: A Descriptive Study in Relation to Day +70 MRD
Source: Int J Mol Sci. 2026 Oct 21;27(20):9348. doi: 10.3390/ijms27209348 (PMC13705239; doi:10.3390/ijms27209348)
Supplement: Supplementary file 1 [file ijms-27-09348-s001.zip › Supplementary Methods.pdf]

Supplementary Methods

Exploratory chromosome-level modelling, reported as a negative result

These analyses are reported here in full, rather than omitted, so that a negative result is not selectively withheld. They are not part of the primary analysis, and no model described below is proposed for clinical use or for the identification of candidate markers.

1. Encoding

Chromosomal involvement was encoded as a binary matrix recording the presence of any mosaic alteration on each of the 24 chromosomes (chr1–22, X, Y) across the 38 MRD-evaluable patients. A chromosome was scored as 1 if the patient carried at least one mosaic copy-number alteration or region of copy-neutral loss of heterozygosity on that chromosome, and 0 otherwise. Day +70 MRD status (positive / negative) was the outcome variable.

2. Models

Two approaches were applied to the same matrix:

- LASSO-penalized logistic regression (glmnet; $\alpha = 1$, λ selected by cross-validated AUC).
- Random-forest classifier (ranger; 1000 trees, permutation importance).

Both models were fitted on a training subset and evaluated on an independent test subset.

3. Results

In the LASSO-penalized model, eight chromosomal features received non-zero coefficients: chr17 ($\beta = 3.38$), chrY ($\beta = 3.28$), chr16 ($\beta = 3.23$) and chr3 ($\beta = 2.74$) in the direction of MRD positivity, and chr9 ($\beta = -1.71$), chr8 ($\beta = -1.16$), chr7 ($\beta = -1.09$) and chrX ($\beta = -0.77$) in the opposite direction. Test ROC AUC was 0.54.

The random-forest classifier ranked chr16, chr6 and chr17 highest by permutation importance, with a test ROC AUC of 0.63.

4. Interpretation

With 13 MRD-positive events against 24 candidate predictors, the events-per-variable ratio is approximately 0.5, against a commonly recommended minimum of ten. Under these conditions, coefficient estimates are expected to be unstable and apparent feature selection is not informative about the underlying association structure. The two approaches retained different features, no significant association with MRD status was identified, and neither model achieved discrimination distinguishable from chance.

Accordingly, the chromosomes retained by these procedures are not reported in the main text as candidate predictive markers, and no inference is drawn from the fitted coefficients.

Table S3. Exploratory chromosome-level models: features retained and discrimination.

**Model**	**Features retained**	**Test ROC AUC**
LASSO	chr17, chrY, chr16, chr3, chr9, chr8, chr7, chrX	0.54
Random forest	chr16, chr6, chr17	0.63

Features retained differed between the two approaches, and neither achieved discrimination distinguishable from chance. Thirteen outcome events against 24 candidate predictors (approximately 0.5 events per variable).
