# Supplementary material for: Copy-Number Alterations and Copy-Neutral Loss of Heterozygosity in Adult T-ALL: A Descriptive Study in Relation to Day +70 MRD
Source: Int J Mol Sci. 2026 Oct 21;27(20):9348. doi: 10.3390/ijms27209348 (PMC13705239; doi:10.3390/ijms27209348)
Supplement: Supplementary file 1 [file ijms-27-09348-s001.zip › Table S1.pdf]

Table S1a. Cytogenetic characterization of MRD-positive patients.

Patient No.	EGIL	ETP	Karyotype	FISH	CMA
1	T - II	non-ETP	46,XY[20]	not revealed MLL/11q23; t(9;22)(q34;q11); del (17p13)/TP53	arr[GRCh38] 13q14.2q14.3(49315464_51854689)x1 [0.62],17q11.2(30615539_32087282)x1 [0.51], 17q21.1q25.3(40060234_83204151)x2 hmz
2	T - I	non-ETP	48,XY,der(3),+4,der(10),der(11),der(19)?t(1;19)(q23;p13),+21[cp3]/46,XY[17]	not revealed t(1;19)(q23;p13); MLL/11q23; PDGFRB/5q32; FGFR1/8p11 10% +one additional signal of gene E2A/19p1; PBX1/1q23; 4q12	arr[GRCh38] 1q21.2q44(149687978_247390677)x3 [0.2],(4)x3 [0.15],(19)x3 [0.2]
3	T - I	ETP	72~76<3n>XY,+X,+Y,der(1),+der(1),?-4,?del(5)(q31),?-7,+10,+11, add(11)(p13-15)x2,add(11)(p15),+12,+21,+22,+mar[cp6]/46,XY[14]	40% +one additional signal of gene TCR A/D/14q11; 40% +one additional signal of gene 17P13/TP53 and centromere 17 not revealed TCR A/D (14q11); del (17p13)/TP53	arr[GRCh38] 1p36.33p35.2(817341_31562811)x1 [0.46],1p35.2q44(31566071_248929896)x3 [0.3],4p16.3q24(20737_101570957)x3 [0.35],4q24q35.2(102258992_190031390)x3 [0.63] , 5q23.3q35.3(128741058_181413624)x1 [0.46],6q14.1(80173957_80439269)x1,(7)x1 [0.5], 8q24.12q24.3(118935412_145076860)x3 [0.35],(9)x1 [0.45],(10)x3 [0.3], 11p15.4q25(3058872_135075654)x3 [0.3],(12)x3 [0.3],(19)x3 [0.3],(20)x3 [0.3],(21)x3 [0.35]
4	T - II	non-ETP	46,XY[20]	not revealed MLL/11q23; t(9;22)(q34;q11); del (17p13)/TP53	arr[GRCh38] 6p25.3p12.3(168801_48979587)x2 hmz,12p13.33p12.3(1391898_16689071)x1 [0.21], 17q11.2(30640847_32080309)x1
5	T - I	ETP	45,X,-Y,del(6)(q22)[9]/45,idem,der(5)[2]/45,X,-Y,del(1)(q3?2),der(6)[cp2]/ 43,X,-Y,t(3;12)(p1?2;p?13),del(6)(q22),-8,-20[1]/46,XY[16]	not revealed MLL/11q23; del (17p13)/TP53	arr[GRCh38] 18q12.2(36189866_36757064)x3
6	T - II	near-ETP	45,XX,t(12;16)(q10;q10)[8]/46,XX [15]	not revealed TCR A/D (14q11); ETV6/12p13 20% one signal is missing from the gene locus ETV6/12p1	arr[GRCh38] 11p11.2p11.12(45860241_50283617)x2 hmz, 12p13.33p13.2(84152_11589326)x1, 12p13.2(11592806_11983676)x0,12p13.2p12.3(11995107_17555050)x1, 12p11.23p11.1(27015648_34716501)x1,16q12.2q24.3(52759610_90086938)x1
7	T - II	near-ETP	46,XX,del(5)(q13q33),del(9)(p11),der(10)?t(10;11)(p12;q21),-11, +mar or der(11)[16]/46,XX[14]	not revealed MLL/11q23	arr[GRCh38] 5q14.3q35.3(85806044_181413624)x1 [0.55], 7q34(142643707_142797442)x1,9p24.2p12(3470883_39588172)x1 [0.55]
8	T - II	non-	46,XX,del(6)(q?21q25),del(7)(q?35),der(	no data	arr[GRCh38] 1p13.2(112467174_113357280)x3

		ETP	11),del(12)(p12)[16]/46,XX[4]		
9	T - III	non-ETP	46,XY,t(11;14)(p32;q11)[18]/46,XX[2]/42,XY,-3,-4,t(4;7)(p?16;q31),-14,-21[1]	not revealed MLL/11q23	arr[GRCh38] 3q23q24(139640691_148507621)x2 hmz,7q34(142623145_142797442)x1, 9p24.3p13.2(382931_36509177)x2 hmz,9p21.3(21834643_21993049)x1
10	T - III	non-ETP	46,XY[24]/46,XY,t(3;9)(q?24;q?31),der(15)[1]	no data	arr[GRCh38] 15q15.1q21.1(42199342_46399090)x2 hmz*
11	T - II	near-ETP	46,XY[20]	50% del PDGFRB/5q32-33	arr[GRCh38] 1p36.33p36.21(817341_15186551)x1,1p36.21p36.13(15192296_17385492)x3, 1p36.13p36.11(19049208_27302054)x3,1p36.11p35.3(27309506_28995610)x1, 1p35.3p35.2(28996797_30598662)x3,1p35.2p33(30937051_48224557)x3, 3q25.32q29(157617797_198173794)x3,5p15.33p14.1(50930_28762095)x2 hmz, 5p14.1p11(28270452_46432814)x3,5q23.3q35.3(129477834_181413624)x1, 6p25.3p22.3(164784_23881712)x3,6q22.31q27(125323457_165120175)x3, 8q21.2q21.3(84347427_91379642)x2 hmz,9p24.3p21.1(204082_32047795)x1, 9p13.3p11.2(33787994_41008635)x1,9q34.12q34.13(130849864_131177358)x3, 12p13.33p12.3(84151_17298766)x1,12q13.13q24.33(54253137_133238370)x2 hmz, 12q24.11q24.11(109110009_109306430)x3,13q13.3q21.2(38762856_59895173)x1 [0.3], 16p13.3p13.3(1729645_3050387)x1,16q24.2q24.3(88544336_89451094)x1, 17p13.3p11.2(142014_19015427)x1,17p11.2q11.2(19238630_30565052)x3, 17q11.2q11.2(30578236_32113996)x1,17q11.2q25.3(32117655_83234813)x3, 19q13.33q13.33(48747281_48933669)x3,19q13.33q13.43(49179734_58586385)x3
12	T - II	non-ETP	43-45,XY, dic(1;?)(1qter->1p?35::?),add(2)(q32),-5,der(5),-7,der(7),?-9, -12, add(11)(q23),-	not revealed MLL/11q23; del (17p13)/TP53 41% +one additional signal of gene	arr[GRCh38] Xp22.33p21.3(2785592_25228099)x2 [0.68], 1p36.33p35.1(817341_32851981)x1 [0.28],1p13.2p11.1(112681793_121888549)x1 [0.27],

			16,add(19)(p?q?13),+3-5 mar,inc [cp15]/46,XY [5]	MLL/11q23	2q36.3q37.3(227102389_241749257)x1 [0.25],3p26.3p26.3(11006_2033129)x3 [0.34], 5p15.1p11(15553525_46432814)x3 [0.43],5q13.2q14.3(71012424_89956785)x3 [0.37], 5q14.3q35.3(89963124_181413624)x1 [0.31],7p22.3p11.2(22117_55953170)x1 [0.31], 7q21.11q34(84864504_142787265)x3 [0.39],8q21.3q22.1(88739768_94206854)x2 hmz, 9q21.11q34.12(68351797_130824565)x1 [0.32],9q34.12q34.13(130824851_131230671)x4, 9q34.13q34.3(131232429_138124244)x1 [0.24],11p11.2p11.12(46072204_50808872)x2 hmz, 11q14.3q25(90848544_135075654)x3 [0.4],12p13.33p11.1(758103_34716501)x1 [0.3], 12q13.13q24.13(53277765_111908857)x1 [0.32],13q22.1q34(72827937_114341910)x3 [0.35], 15q11.2q15.1(22651951_41924490)x1 [0.36],16p13.3p11.2(181763_29169656)x1 [0.3], 16q11.2q21(46381628_62609027)x3 [0.38],16q21q24.3(62616583_90086938)x1 [0.29], 17q11.2q21.2(32062643_40907982)x1 [0.27],22q13.2q13.33(41818234_50759324)x1 [0.26]
13	Mixed (T-Myelo)	ETP	46,XX[5]	no data	arr[GRCh38] Xq26.2q26.3(134158868_135170305)x1,6p25.3p21.2(164785_37915004)x3, 7p22.3p11.2(22118_55423221)x1

FISH — fluorescence in situ hybridization; CMA — chromosomal microarray analysis; MRD — measurable (minimal) residual disease.

*Regions of cnLOH of 3 Mb or more identified on re-analysis of the raw CMA data; the clinical laboratory report for this patient was normal.

Table S1b. Cytogenetic characterization of MRD-negative patients.

Patient No.	EGIL	ETP	Karyotype	FISH	CMA
14	T - I	ETP	48,XY,der(17),+2mar,inc[cp3]/47,XY ,-9,der(17),+2mar,inc[cp5]	not revealed MLL/11q23; del (17p13)/TP53; BCL11B/14q32.2	arr[GRCh38] (4)x3 [0.6],6p23p22.3(13666060_16427293)x1, 7p11.2q11.22(54487403_69079311) x2 hmz,7q11.23q36.3(73732738_159332308)x2 hmz,

					8p23.3p12(214984_32712975)x1 [0.2],9q21.11q31.1(68351797_104628316)x1 [0.2], 13q22.1q31.1(73726388_78827304)x2 hmz,15q13.2q13.3(30821122_32139875)x3, 21q21.2q22.3(24719287_46670717)x3 [0.2]
15	T - II	non-ETP	47,XY,+16[18]/46,XY[2]	no data	arr[GRCh38] 7q34(142659319_142785320)x0, 9p24.3p13.3(204082_33290534)x2 hmz,9p21.3(21682143_22103814)x0,(16)x3
16	T - III	non-ETP	46,XY[25]	not revealed MLL/11q23;	arr[GRCh38] 9q33.1(116635278_116805429)x1,10q21.1q21.1(55254465_58745172)x2 hmz
17	T - III	non-ETP	46,XY,t(10;14)(q24;q11)[11]/47,idem,+8[8]/46,XY[1]	no data	arr[GRCh38] 1p31.3(65421924_65462140)x1, 1q22(155232726_155449495)x1,2p22.3p16.3(35980818_50313647)x2 hmz, 2p12p11.2(80482793_85686091)x2 hmz,2q12.1q14.1(104484671_115003909)x2 hmz, 6p22.3p22.1(22886608_27897130)x2 hmz,7q34(142637680_142796623)x0 (8)x3 [0.39],9p21.3(21471728_22313094)x0,9q34.12q34.13(130849865_131217919)x3, 10q22.1q22.3(71580364_75914747)x2 hmz,13q12.13q12.3(25950828_29969779)x2 hmz, 13q14.2(48411504_48496038)x0,14q31.1q31.3(80499962_87760886)x2 hmz
18	T - III	non-ETP	47,XX,t(7;7)(p15;q34),+9,del(9)(p2?1)x2[7]/47,XX,t(7;7)(p15;q34),+9,del(9)(p2?1)x2,?del(12)(p1?3)[1]/46,XX[12]	no data	arr[GRCh38] 1q31.1q31.3(188660626_195902787)x2 hmz,9p24.3p21.3(204082_20631634)x3 [0.2], 9p21.3p12(20638249_39588172)x1 [0.23],9q21.11q34.3(69129174_138124244)x3 [0.2], 16q12.13q13.1(25960854_32998442)x2 hmz,16 p12.1p11.2(28479196_31935920)x2 hmz
19	T - III	non-ETP	46,XX,t(1;14)(p32;q11)[cp11]/47,idem,+7[cp4]	no data	arr[GRCh38] 7p22.3q34(201901_142433163)x3 [0.3],7q34(142438198_142787265)x0, 7q34q36.3(142787265_159334770)x3 [0.3],9p24.3p13.3(204082_33414186)x2 hmz
20	T - II	non-ETP	46,XY[20]	not revealed MLL/11q23;	arr[GRCh38] 7q34(142630162_142796623)x1,

				del (17p13)/TP53	9p24.3p13.3(204082_35880777)x2 hmz,11p11.2p11.12(46288509_50808872)x2 hmz
21	Mixed (T-Myelo)	ETP	46,XX[20]	no data	arr[GRCh38] (4)x3 [0.15], 9p24.3p13.3(204082_34203681)x2 hmz,9p21.3(21940589_21980152)x1, 11q14.2q14.3(88575341_91645792)x2 hmz
22	T - III	non-ETP	46,XY[20]	no data	arr(X,Y)x1,(1-22)x2
23	T - III	non-ETP	no data	no data	arr[GRCh38] 1p36.12p36.11(22764780_24293941)x1 [0.4],9p21.3(21677460_23358877)x1, 12p13.2p13.1(10687083_14521433)x1 [0.4]
24	T - III	non-ETP	46,XY,t(6;7)(q23;q34)[20]/46,XY[10]	not revealed MLL/11q23	arr[GRCh38] 2p25.1(8998132_9693497)x1, 3p21.31p21.1(45791067_53845345)x2 hmz,7q34(142309952_142795256)x0, 8q23.3q24.11(116631907_117128872)x1,9p22.1p21.3(19703574_20908514)x1, 9p21.3(20914896_22056500)x0,9p21.3(22061616_25458801)x1, 15q15.1q21.1(42134595_45151566)x2 hmz
25	T - IV	non-ETP	46,XY[12]	not revealed IGH/14q32; MLL/11q23; del (17p13)/TP53; TCR A/D/14q11	arr[GRCh38] 6q23.3(135157728_135394832)x3,7q34(142342129_142785320)x1, 9p24.3p13.1(204082_38771834)x2 hmz
26	T - II	non-ETP	47,XY,t(10;11)(p12;q21),+13[15]/46,XY[5]	not revealed MLL/11q23	arr[GRCh38] 1p36.22p32.2(10875175_56330265)x2 hmz, 2q14.2q14.3(118979389_128439038)x2 hmz, 3p24.3p23(18163160_31734262)x2 hmz, 3q13.11q13.31(105939348_114977458)x2 hmz, 3q21.1q22.2(123192949_135549353)x2 hmz, 5q35.2q35.3(173355575_181288220)x2 hmz, 7q35q36.1(145999413_151017812)x2 hmz, 7q36.3q36.3(155936328_159332308)x2 hmz, 8p23.3p22(1682303_16134616)x2 hmz, 9p24.3p13.3(204082_33983729)x2 hmz, 9q21.33q31.3(87677710_109245885)x2 hmz, 12p13.2q13.13(10630453_54471741)x2 hmz,12q23.3q24.22(108299453_117692135)x2 hmz, 13q11q14.2(18865955_48012163)x3,13q14.3q34(51859429_114341910)x3, 13q14.3q21.31(51154071_63628328)x2 hmz, 14q32.2q32.33(98428116_106879456)x2 hmz,

					16q22.2q23.1(71434323_78927286)x2 hmz, 17p13.3p13.2(151041_6123247)x2 hmz, 18p11.32p11.21(132649_11878597)x2 hmz, 19q13.32q13.41(46667387_52212463)x2 hmz,20q13.31q13.33(56532426_61716605)x2 hmz
27	T - II	ETP	47,XY,t(1;11)(p32;q23),del(9)(p21),i(9)(q10),add(10)(p15),+mar[11]/48,idem,del(2)(q23),+mar2[1]/ 46,XY[8]	not revealed MLL/11q23; t(9;22)(q34;q11); 2% +one additional signal of gene ABL (9q34)	arr[GRCh38] 3q25.33q26.1(160673803_164562696)x2 hmz,5p15.33p12(11983_43658409)x4, 8p23.1(11522557_11601509)x3,9p24.3p21.3(204083_21635941)x1 [0.76], 9p21.3(20272224_22647015)x0 [0.5],9p21.3p13.1(22681778_38780194)x1 [0.76], 9q21.11q34.3(68351798_138124244)x3,10p15.1p14(5935973_9688461)x1 [0.74] 19p13.3p13.12(3771113_15026349)x2 hmz
28	T - III	non-ETP	46,XY[20]	not revealed MLL/11q23; del (17p13)/TP53	arr[GRCh38] 4p12(45520568_46381622)x3, 5q11.2q(54030404_57198151)x2 hmz,7p22.3(143569_267679)x3
29	T - III	non-ETP	no data	not revealed MLL/11q23	arr[GRCh38] Yq11.21q11.221(12384958_12855767)x2,5q14.1q35.3(79332410_181288220)x2 hmz, 7q34(142610489_142796623)x0,9p21.3(21972814_22025494)x0, 12p13.31p13.1(7656831_13999631)x1
30	T - III	non-ETP	no data	not revealed MLL/11q23	arr[GRCh38] Xp22.33p22.31(249587_8241004)x3 [0.23], 1q42.2q43(232219946_237329914)x2 hmz,2p16.2p14(54441063_66063380)x2 hmz, 3p22.3p21.31(32126001_46668608)x2 hmz,3q21.1q21.3(123643056_127819496)x2 hmz, 3q26.1q26.2(166020100_169271386)x2 hmz,4p15.33p15.32(12823770_16445690)x2 hmz, 5q23.1q23.2(121569455_126383675)x2 hmz,7q22.3(105078413_105204647)x1, 7q34(142620585_142795256)x1, 9p21.3(21052665_21865843)x1,9p21.3(21866502_21996864)x0, 9p21.3(21998037_25552602)x1,11p13(32406817_32581629)x1, 11p11.2p11.12(46155161_57606859)x2 hmz,12q21.31q21.32(84793732_88408683)x2 hmz,

					16p12.3p12.1(20668830_26796982)x2 hmz,17q22q24.2(58307366_66751216)x2 hmz
31	T - III	non-ETP	45,XY,der(3)t(3;9)(p?10;q?10),-9[14]/92,idem[4]/46,XY[2]	not revealed t(9;22)(q34;q11);<br>20% tetraploid in gene loci ABL/9q34 and BCR/22q11;	arr[GRCh38] 11p14.3p13(25652487_32032509)x2 hmz,16q21q21(61053988_66378753)x2 hmz, 18q11.1q11.2(20948500_24208783)x2 hmz
32	Mixed (T-Myelo)	ETP	46,XY[20]	not revealed t(9;22)(q34;q11);	arr[GRCh38] 6q25.3(156205949_157032319)x1
33	T - III	non-ETP	46,XX[15]	no data	arr[GRCh38] 1p22.1 (92596464_93028067)x1,2q32.2 (188750266_189163011)x1, 7q11.21(63841784_67080877)x2 hmz,7q34q36.3(142318162_155489259)x1, 7q36.3(158574469_158839379)x3,9p24.3p21.3(204082_21905380)x1,9p21.3(21909980_22233512)x0, 9p21.3p11.2(22238596_40880243)x1,11q21(95764067_95823093)x1,15q24.1(73659794_75166509)x1, 16p12.2(21323145_21897322)x3,17p13.1(10071980_10512523)x3, 19p13.2p13.13(10992086_12754890)x3,19p13.13p12(12761900_20470790)x4, 19p12q13.12(20557343_37537677)x3,19q13.2(38294454_39428898)x3, 20q13.33(63293072_64033000)x1
34	T - II	non-ETP	46,XY[11]	no data	arr[GRCh38] 1q32.1q44(199241828_248929896)x3,7q34(142538468_142787265)x0, 7q34q36.1(142792254_151451166)x1,12q13.11q24.33(47932060_133238370)x2 hmz, 13q12.3q34(29165142_114341035)x2 hmz
35	T - II	non-ETP	46,XX,der(5),der(8),del(9)(p23),del(12)(p11),der(17)[10]/46,XX[20]	not revealed MLL/11q23; t(9;22)(q34;q11); del (17p13)/TP53<br>57% no signal from the gene locus ABL/9q34	arr[GRCh38] 3q22.2q23(135208501_141168937)x2 hmz,5q11.2q13.3(53021318_75340775)x1 [0.6], 5q21.2q22.1(104459991_111045559)x1 [0.66],5q31.1(134161253_134738580)x1 [0.6], 8p22(14892962_16191290)x1 [0.59],8q23.2q23.3(109541533_115522093)x1 [0.62], 9q34.11q34.13(128685225_131157056)x1 [0.63],12p13.31p11.1(8858660_34716501)x1 [0.65], 16p13.11(15046800_16242519)x3,16q12.1q22.1(49550309_69

					998036)x1 [0.6], 17q11.2q12(30957012_36149761)x1 [0.57],17q24.2q25.1(68223392_73711840)x1 [0.66]]
36	T - II	near-ETP	46,XX[20]	no data	arr[GRCh38] 1p33(47233031_47312561)x1, 5p15.33p13.1(11982_41119133)x3 [0.22],6q22.1q22.31(114210484_124599994)x2 hmz, 7q34(142480213_142787265)x1,9p24.3p21.1(204082_29257578)x1 [0.23], 15q15.1q21.1(42009696_45139857)x2 hmz
37	T - I	ETP	46,XY[20]	no data	arr[GRCh38] 1q24.3q25.1(172088643_175545039)x2 hmz,2q11.2(96978119_100123597)x2 hmz, 3p21.31p21.1(47926580_52307110)x2 hmz,7q31.1q31.2(112157492_115902231)x2 hmz, 8q23.3q24.11(114784950_117846798)x2 hmz,11q13.4q13.5(72263152_77092402)x2 hmz, 12q24.11q24.13(109818520_113515908)x2 hmz,14q23.2q24.1(64109344_67505168)x2 hmz, 19p13.11p11(18854146_24324012)x2 hmz*
38	T - I	ETP	46, XY, t(6;14)(q25;q21) [20]	not revealed t(9;22)(q34;q11);	arr[GRCh38] 4q31.1q35.2(139333364_190031390)x3 [0.15], 7q34q36.2(138805138_154804306)x1 [0.17],8q23.1q24.13(107756162_125656284)x1 [0.13], 9q21.32q22.32(82630060_95953754)x1 [0.15],10q21.3q22.3(64750138_80038618)x2 hmz, 10q26.13q26.3(123319796_128934882)x2 hmz,11q14.3q21(91399323_94975200)x2 hmz, 19q12q13.2(29264977_38315691)x2 hmz

FISH — fluorescence in situ hybridization; CMA — chromosomal microarray analysis; MRD — measurable (minimal) residual disease.

*Regions of cnLOH of 3 Mb or more identified on re-analysis of the raw CMA data; the clinical laboratory report for this patient was normal.

Table S1c. Cytogenetic characterization of a patient with early mortality (MRD not determined).

Patient No.	EGIL	ETP	Karyotype	FISH	CMA
39	T - II	near-ETP	82~93<4n>,XXXX,+3,-7,-7,der(9)x2,?+15,+16,der(17),+2mar[cp11]/ 90<4n>,XXX,-X,-1,der(1),-	not revealed MLL/11q23; t(9;22)(q34;q11); TCR A/D (14q11)	arr[GRCh38] (X)x1 [0.6],(4)x3 [0.5],5p15.33p11(11982_46432814)x3 [0.4], 5q11.2q35.3(52541105_181413624)x1 [0.5],(6)x1 [0.1],(7)x1

			2,der(3),dic(3;?)(p?;?),der(5),-7,-7,der(9),add(11)(p?), add(12)(q?),-13,-14,der(17),-19,?der(19),+7mar[1]/46,XX[4]	90% nuclei revealed tetrasomy 17 with a del (17p13)/TP53 of one chromosome; tetrasomy 11 and 14;<br>90% +one-four additional signal of gene ABL/9q34 and BCR/22q11	[0.4],(10)x3 [0.2],(13)x3 [0.4], 17p13.3p11.2(142014_19248289)x1 [0.42],17p11.2q11.2(19254773_30633042)x3 [0.5], 17q11.2(30636678_32062643)x1,17q11.2q25.3(32069579_83234813)x3 [0.5]

FISH — fluorescence in situ hybridization; CMA — chromosomal microarray analysis; MRD — measurable (minimal) residual disease.
